# Effects of clinical stage, behavioral and psychological symptoms of dementia, and living arrangement on social distance towards people with dementia

**DOI:** 10.1371/journal.pone.0317911

**Published:** 2025-01-22

**Authors:** Kae Ito, Shuji Tsuda

**Affiliations:** Tokyo Metropolitan Institute for Geriatrics and Gerontology, Tokyo, Japan; Ehime University Graduate School of Medicine, JAPAN

## Abstract

**Background:**

Dementia presents significant challenges, including social exclusion, which can be exacerbated by public stigma. This study aimed to clarify how social distances, a common measure of public stigma, towards people living with dementia and its associated factors vary with clinical stage, presence of behavioral and psychological symptoms of dementia (BPSD), and living arrangements.

**Methods:**

The study involved 2,589 Japanese participants aged 40 to 90 years. They were exposed to one of four vignettes depicting an 80-year-old woman progressing from normal ageing to mild, moderate, and severe dementia: Vignette A (living with husband, without BPSD); Vignette B (living with husband, with BPSD); Vignette C (living alone, without BPSD); and Vignette D (living alone, with BPSD).

**Results:**

Social distance showed no significant differences in the normal aging and mild stage of dementia across all vignettes. At the moderate stage, social distance was higher for individuals exhibiting BPSD, regardless of living arrangement. At the severe stage, the lowest social distance was observed towards individuals living with their family without BPSD, whereas the highest was towards those living alone, exhibiting BPSD. For Vignette A, possession of social capital (p<0.001) and having experience of social contact with people living with dementia (PLWD) (p = 0.001–0.007) were independently associated with lower social distance across all dementia stages. In addition, in the mild stage of dementia, high perceived social support (p = 0.005) and having knowledge about dementia (p = 0.036) were associated with lower social distance, but not in the moderate or severe stage of dementia. For Vignette D, possession of social capital (p≤0.001) and having experience of social contact with PLWD (p<0.001 to p = 0.006) were independently associated with lower social distance across all dementia stages. In mild dementia, female sex (p = 0.004) and knowledge about dementia (p = 0.026) were associated with lower social distance. Furthermore, in mild and moderate dementia, living in rural area (p = 0.003–0.048) was associated with lower social distance.

**Conclusions:**

Social distance is higher toward PLWD who live alone and exhibit BPSD than toward those who live with family and/or do not show BPSD, indicating a higher risk of exclusion for the former. Moreover, factors affecting social distance towards PLWD vary across different clinical stages of dementia. While greater knowledge about dementia is associated with lower social distance toward PLWD, this effect appears to be most pronounced in the mild stage. In contrast, opportunities for social contact with PLWD are crucial for achieving lower social distance across all stages of dementia. The findings underscore the need for stage-specific interventions to address stigma, with a focus on education and opportunities for social contact. Targeted efforts are especially important for promoting the inclusion of PLWD who live alone and exhibit BPSD.

## Background

Currently, more than 55 million people worldwide are living with dementia, and this number is projected to increase by 10 million annually [1]. By 2050, the population of persons living with dementia (PLWD) is expected to exceed 139 million [2]. PLWD and their informal caregivers face challenges including stigma and social exclusion [3]. In some cases, the burden of stigma outweighs that of the condition itself [4, 5]. Stigma has been linked to adverse outcomes for people with mental health conditions, acting as a barrier to help-seeking and the achievement of age-appropriate functional goals [6]. It affects people psychologically, restricts social participation, and creates barriers to maintaining a good quality of life [6, 7]. Some researchers argue that stigma constitutes a form of societal discrimination and can be considered a human rights violation [8].

### Definition of stigma

Goffman [9], who first introduced the term stigma in sociology, defined it as “the situation of the individual who is disqualified from full social acceptance.” He emphasized that stigma arises from social interactions rather than individual attributes, suggesting a need for language focused on relationships. Stigma comprises stereotyping, prejudice, and discrimination: stereotyping as the cognitive component, prejudice as the affective component, and discrimination as the behavioral component [10].

Link and Phelan defined stigma as the co-occurrence of labeling, stereotyping, separation, status loss, discrimination, and the power dynamics that enable these components [11]. Mental health conditions stigma has been distinguished by three relevant dimensions: public stigma, self-stigma, and institutional stigma. According to the American Psychiatric Association [12], public stigma refers to the negative or discriminatory attitudes others may have about mental illness. Self-stigma involves the negative attitudes, including internalized shame, that people with psychiatric disorders may have about their own condition. Structural stigma is systemic, involving policies of government and private organizations that intentionally or unintentionally limit opportunities for people with mental health conditions, such as lower funding for mental health condition research or fewer mental health services relative to other healthcare. Thornicroft [5] added stigma by association, where family members face negative stereotypes and discrimination due to their association with PLWD.

### Public stigma and social distance

Effective interventions to reduce stigma often focus on public stigma [5], viewed as a broad societal force affecting both individuals and society [13]. One significant source of stigma is community members [14], and the debilitating effects of Alzheimer’s disease (AD) may be exacerbated by public stigma [15, 16]. Social distance is a common measure of public stigma [7, 17, 18] reflecting the desired proximity or “social distancing” from others’ experiences and circumstances [19]. Park [20] initially defined social distance as “the grades and degrees of understanding and intimacy which characterize pre-social and social relations generally.” In stigma research, it is defined as the desired degree of closeness or distancing from another individual [21], conceptualized as the behavioral consequence of stigma [22].

### Experimental vignette studies

After Scheff’s discussion [23] on the labeling of mental health conditions, numerous studies have examined the impact of stigmatization on individuals with such conditions [13]. Of them, research specifically targeting dementia-related stigma is increasing [24]. What is rather specific about dementia is that dementia often progresses through a latent period that is difficult to distinguish from normal aging, with approximately 90% of the patients experiencing behavioral and psychological symptoms of dementia (BPSD) at some point [25]. Public perceptions of PLWD vary based on personal experiences. Experimental vignette methodology [26] helps capture stigma against individuals depicted in vignettes, not the diverse and sometimes inaccurate images of dementia that individuals have. A previous study [27] showed that participants exposed to vignettes depicting a person with dementia expressed lower levels of stigma than those not exposed.

The studies that applied experimental vignettes to explore dementia-related stigma have shown that the severity of dementia affects social distance [28, 29], and perceptions of dangerousness, fear, and anger affect discriminatory behavior [16]. Other studies have demonstrated that stereotypes, prejudice, and discrimination are prevalent in relation to AD [30], and the worsening prognosis of dementia is associated with stronger discrimination and larger social distance [31]. However, despite the progressive nature of dementia, few studies have explored how social distance changes as dementia progresses. One notable example is a vignette study by Stites et al., which compared individuals with no dementia, mild Alzheimer’s disease (AD), and moderate AD. The study found that both mild and moderate AD were associated with greater stigma compared to no dementia [32].

### Aim of the study

This study aimed to clarify variations in social distance, a common measure of public stigma, towards PLWD and its associated factors. Specifically, it investigated how clinical stage, presence of BPSD, and living arrangements influence social distance, using an experimental vignette methodology [26].

## Methods

### Participants

The participants were 2589 community-dwelling men and women in Japan, aged 40 to 90 years, who registered with an online survey company.

### Procedure

The social distance scale uses sex-specific, condition-adjusted vignettes that describe a person with typical features of the condition. Given that depictions of dementia in popular culture often focus on AD [33], that AD is the most prevalent dementia disease in Japan, accounting for 67.6% of all cases [34], and considering that the morbidity rate for women is higher than for men [34], a woman with AD was depicted as the typical feature of dementia.

After giving consent online, participants were exposed to vignettes describing an 80-year-old woman with AD. Four versions of the vignettes were designed, and each participant received one vignette (see S1 Appendix). The vignettes varied by 1) living arrangement and 2) the presence of behavioral and psychological symptoms of dementia (BPSD): Vignette A depicted Ms. A, who lives with her husband and does not exhibit BPSD; Vignette B described Ms. B, who lives with her husband, and shows BPSD; Vignette C depicted Ms. C, who lives alone, and does not show BPSD; and Vignette D described Ms. D, who lives alone and shows BPSD. Each vignette illustrated a woman’s progression from normal aging to mild, moderate, and severe dementia. The vignettes were designed to meet the diagnostic criteria for dementia outlined in the Diagnostic and Statistical Manual of Mental Disorders, Fifth Edition (DSM-5) [35]. The clinical stages of dementia in the vignettes were defined using the Clinical Dementia Rating (CDR) [36, 37] and Functional Assessment Staging of Alzheimer’s Disease (FAST) [38]: normal aging (CDR = 0, FAST 2), mild dementia (CDR = 1, FAST 4), moderate dementia (CDR = 2, FAST 5), and severe dementia (CDR = 3, FAST 6a, b).

The vignettes were initially developed by the authors and subsequently refined with input from an experienced geriatric-psychiatrist specializing in dementia. To enhance the realism and maintain neutrality in the presentation, crucial since the degree of stigma can be influenced by the scenario’s portrayal [39], two pairs of individuals diagnosed with AD and their caregivers reviewed the vignettes and checked their reality and neutrality.

Each version of the vignettes was sequentially assigned to participants, balancing for factors such as age, sex, and living area. This process continued until the registration closed, with a total of 640 responses received for each vignette version (Table 1).

**Table 1:** Vignette assignment.

		Vignette A				Vignette B				Vignette C				Vignette D			
	Living arrangement	Not living alone								Living alone							
	BPSD	Not present				Present				Not present				Present			
Rural area	Age, y	<65		≥65		<65		≥65		<65		≥65		<65		≥65	
	Sex	M	F	M	F	M	F	M	F	M	F	M	F	M	F	M	F
	n	79	83	82	82	79	77	83	83	76	81	85	81	80	74	83	80
Urban area	Age, y	<65		≥65		<65		≥65		<65		≥65		<65		≥65	
	Sex	M	F	M	F	M	F	M	F	M	F	M	F	M	F	M	F
	n	81	78	81	81	78	81	80	79	83	85	89	80	81	80	79	83
Total number of participants per vignette					647				640				660				640

Abbreviations: BPSD, behavioral and psychological symptoms of dementia; M, male; F, female

Each version of the vignettes was sequentially assigned to participants, balancing for age, sex, and living area.

#### Ethical considerations

All the methods and procedures carried out in this study were in accordance with relevant guidelines and regulation. The online survey started after the aim of the study was explained in written form to the participants, and consent was obtained online. Ethical approval was obtained from the Institutional Review Board and Ethics Committee of Tokyo Metropolitan Institute of Gerontology (Approval No. R23-109).

#### Funding

This study was supported by Health Labour Science Research Grant by the Japanese Ministry of Health, Labour and Welfare (No. 22GB1003). The funders had no role in study design, data collection and analysis, decision to publish, or preparation of the manuscript. None of the authors have any commercial or financial involvements in connection with this study that represent or appear to represent any conflicts of interest.

### Measures

The questionnaire included items concerning social distance and background characteristics.

#### Social distance

Similar to Werner [28], to measure social distance, participants were asked to respond to four items regarding their willingness to engage with the person depicted in the vignette: participants were asked how willing they would be to (1) live near the person depicted in the vignette; (2) spend an evening socializing with the person; (3) make friends with the person; and (4) have their child or grandchild marry the person’s child or grandchild. Each item was rated on a five-point Likert-type scale ranging from 0 (definitely willing) to 4 (definitely unwilling). The scores for these four items were summed (social distance score), with higher scores indicating greater social distance.

#### Background characteristics

Background characteristics included sociodemographic factors, health-related factors, dementia exposure and knowledge, and cultural perspectives.

Sociodemographic factors: Sociodemographic factors included age, sex, years of education, marital status, living area, living arrangement, family income per year, social capital, and perceived social support. Years of education were categorized into three groups: ≤9 years (compulsory education), 10–12 years (senior high school), and ≥13 years (beyond senior high school). Marital Status was assessed with the question, “Are you married?”, with possible answers “yes” or “no.” Living area was categorized as urban or rural, with rural areas designated as depopulated by the Ministry of Internal Affairs and Communications, and urban areas as others. Living arrangement was categorized as living alone or not living alone. Family income was categorized as above average or below average based on the 2021 average family income in Japan (5,500,000yen, equivalent to about 33,000dollars). Social capital (SC) was evaluated with five questions: “Do you think people living in your community are generally trustworthy?” (SC1), “Do you think people living in your community often try to help others?” (SC2), and “How attached are you to the community in which you currently live?” (SC3). Each item was rated on a five-point Likert-type scale ranging from 1 = definitely to 5 = definitely not. Responses of 1 and 2 were categorized as 1 (possess), and 3, 4, and 5 were categorized as 0 (do not possess), and then summed. Perceived social support (PSS) was evaluated with three questions with “yes” or “no” responses: “Do you have someone whom you can talk to about your concerns and complaints?” (PSS1), “Does someone talk to you about their concerns and complaints?” (PSS2), “Do you have someone who would take care of you when you are ill in bed for a few days?” (PSS3). Each item was binarized into 1(present) and 0(not present), then summed.

Health-related factors: Subjective perception of health was assessed by the question, “How would you describe your current overall health?” Possible answers were 1 (very good), 2 (fairly good), 3 (not good), and 4 (bad). Responses 1 and 2 were categorized as 1 (good), and 3,4 as 0 (bad).

Dementia exposure and knowledge: Knowledge was assessed by the Scale to Assess Dementia-Related Knowledge among Local Residents [40], with higher scores indicating having more knowledge (score range: 0 to 10). Experience of learning about dementia (learning about dementia) was assessed with the question, “Have you ever had an experience of learning about dementia?” with possible answers “yes” or “no.” Experience of social contact with PLWD (social contact with PLWD) was assessed with the question, “Have you ever had an experience of social contact with PLWD?” with possible answers “yes” or “no.”

Cultural perspectives: Cultural perspectives were assessed by the short version of Scale for Measuring Independent and Interdependent Construal of Self [41, 42]. Markus and Kitayama [43] proposed that individuals in different cultures have distinctly different construals of the self, which can influence cognition, emotion, and motivation. This scale measures the extent to which an individual’s self-construal reflects independent and interdependent construals. It includes 10 items, with four items measuring independent construal and six items measuring interdependent construal. Independent construal consists of “self-confidence/ assertiveness” (2 items) and “strong personal conviction” (2 items), whereas interdependent construal consists of “social harmony/adaption” (4 items) and “evaluation concerns” (2 items). Each item is assessed using a seven-point Likert scale. Independent construals are related to individualism, and interdependent construals are related to collectivism.

### Statistical analysis

Statistical analyses were performed using SPSS Statistics version 29.0.1.0 for Windows (IBM). Differences in social distance according to vignette and clinical stage of dementia were examined using Bonferroni multiple comparisons. To investigate the associations between social distance and key variables, multivariable linear regression analysis was conducted for each of the three clinical stages of dementia (mild, moderate, and severe) for each vignette (A–D). The results were reported as regression coefficients (β), 95% confidence intervals (CIs), and p-values, with a significance level of p < 0.05. The analysis was performed separately for each vignette and by clinical stage of dementia. To explore how the roles of associated factors varied across clinical stages, detailed factor-based analyses were conducted. This approach focused on factors that meet all three criteria: 1) factors associated with social distance toward PLWD identified through multivariable regression analysis in the present study, 2) factors previously identified as associated with social distance toward PLWD, and 3) factors whose effects on social distance were found to vary depending on the dementia stage in the present study. Rather than conducting new analyses, this method provided a more detailed understanding of how specific factors influence social distance at each stage of dementia.

As this is a cross-sectional observational study, the analyses focus on describing differences between groups at a single point in time. The results do not imply causal relationships or longitudinal effects. For factors consistently associated with social distance across all dementia stages, only p-values are reported. For factors with stage-specific associations, both 95% confidence intervals (CIs) and p-values are presented to illustrate the magnitude and precision of the effect.

## Results

### Participants’ characteristics

A total of 2589 participants aged 40 to 90 years completed the survey. Table 2 (placed at the end of the document) presents the characteristics of the participants. The average age ± standard deviation (SD) was 62.0±10.5 years (range: 41 to 91 years). The male-to-female ratio was 50.2: 49.8, and 72.4% were married. In terms of education, 5.5% had less than 9 years, 37.9% had 10 to 12 years, and 56.5% had more than 13 years of education. Of the participants, 19.4% were living alone, and 62.1% had an income below the average. In addition, 28.7% had experience of learning about dementia, and 63.2% had experience of contact with PLWD. The mean score ± SD for the Scale to Assess Dementia-Related Knowledge among Local Residents was 4.9±1.7. For reference, the mean score ± SD in the previous study conducted by the developers of this scale on community residents was 4.9±2.6 [40]. Analysis of variance showed no sample bias across the vignettes for any of the items.

**Table 2:** Participants’ characteristics (n = 2589).

		Total		Vignette A		Vignette B		Vignette C		Vignette D		
Socio-demographic characteristics		n	%	n	%	n	%	n	%	n	%	p
Age, y		mean ± SD	62.0±10.5	62.1±10.6		61.8±10.5		62.1±10.6		61.8±10.5		0.950
Sex	Male	1299	50.2	323	49.8	320	50.0	333	50.5	323	50.4	0.995
	Female	1290	49.8	325	50.2	320	50.0	327	49.5	318	49.6	
Years of education	≥13	1463	56.6	364	56.2	376	58.9	371	56.3	352	54.9	0.260
	10–12	981	37.9	242	37.3	238	37.3	248	3706	253	39.5	
	≤9	142	5.5	42	6.5	24	3.8	40	6.1	36	5.6	
Marital status	Married	1874	72.4	454	70.1	461	72.0	501	75.9	458	71.5	0.104
	Not married	715	27.6	194	29.9	179	28.0	159	24.1	183	28.5	
Living area	Urban	1299	50.2	321	49.6	318	49.7	337	51.1	323	50.5	0.946
	Rural	1288	49.7	326	50.4	322	50.3	323	48.9	317	49.5	
Living arrangement	Living alone	503	19.4	134	20.7	119	18.6	115	17.4	135	21.1	0.294
	Not living alone	2086	80.6	514	79.3	521	81.4	545	82.6	506	78.9	
Family income per year	Above average	980	37.5	247	38.4	244	38.1	253	38.3	234	36.5	0.859
	Below average	1609	62.1	399	61.6	396	61.9	407	61.7	407	63.5	
Social capital	(0–3)	mean ± SD	1.6±1.2	1.6±1.2		1.7±1.2		1.6±1.2		1.7±1.2		0.175
Perceived social support	(0–3)	mean ± SD	2.6±0.8	2.6±0.8		2.7±0.7		2.7±0.8		2.6±0.9		0.071
**Health-Related Factor**		n	%									
Subjective health	good	2048	79.1	522	80.6	519	81.1	511	77.4	496	77.4	0.200
	bad	541	20.9	126	19.4	121	18.9	149	22.6	145	22.6	
**Dementia exposure and knowledge**		n	%									
Knowledge about dementia	(0–10)	mean ± SD	4.9±1.7	4.9±1.7		5.0±1.7		4.9±1.8		4.9±1.7		0.831
Experience of learning about dementia	present	742	28.7	178	27.5	185	28.9	188	28.5	191	29.8	0.830
Experience of social contact with PLWD	present	1637	63.2	414	63.9	398	62.2	413	62.6	412	64.3	0.840
**Cultural perspectives**												
Self-confidence/ assertiveness	(0–12)	mean ± SD	6.3±2.1	6.4±2.1		6.3±2.2		6.4±2.0		6.2±2.1		0.32
Strong personal conviction	(0–12)	mean ± SD	6.9±2.1	7.0±2.1		6.9±2.1		6.9±1.9		6.8±2.2		0.409
Social harmony/adaption	(0–24)	mean ± SD	13.7±3.0	13.8±3.0		13.6±3.1		13.8±2.6		13.7±3.1		0.427
Evaluation concerns	(0–12)	mean ± SD	6.1±2.7	6.1±2.9		6.1±2.7		6.4±2.0		6.2±2.1		0.069

Abbreviations: PLWD, people living with dementia

### Vignette-specific changes in social distance by clinical stage of dementia

**Fig 1:**
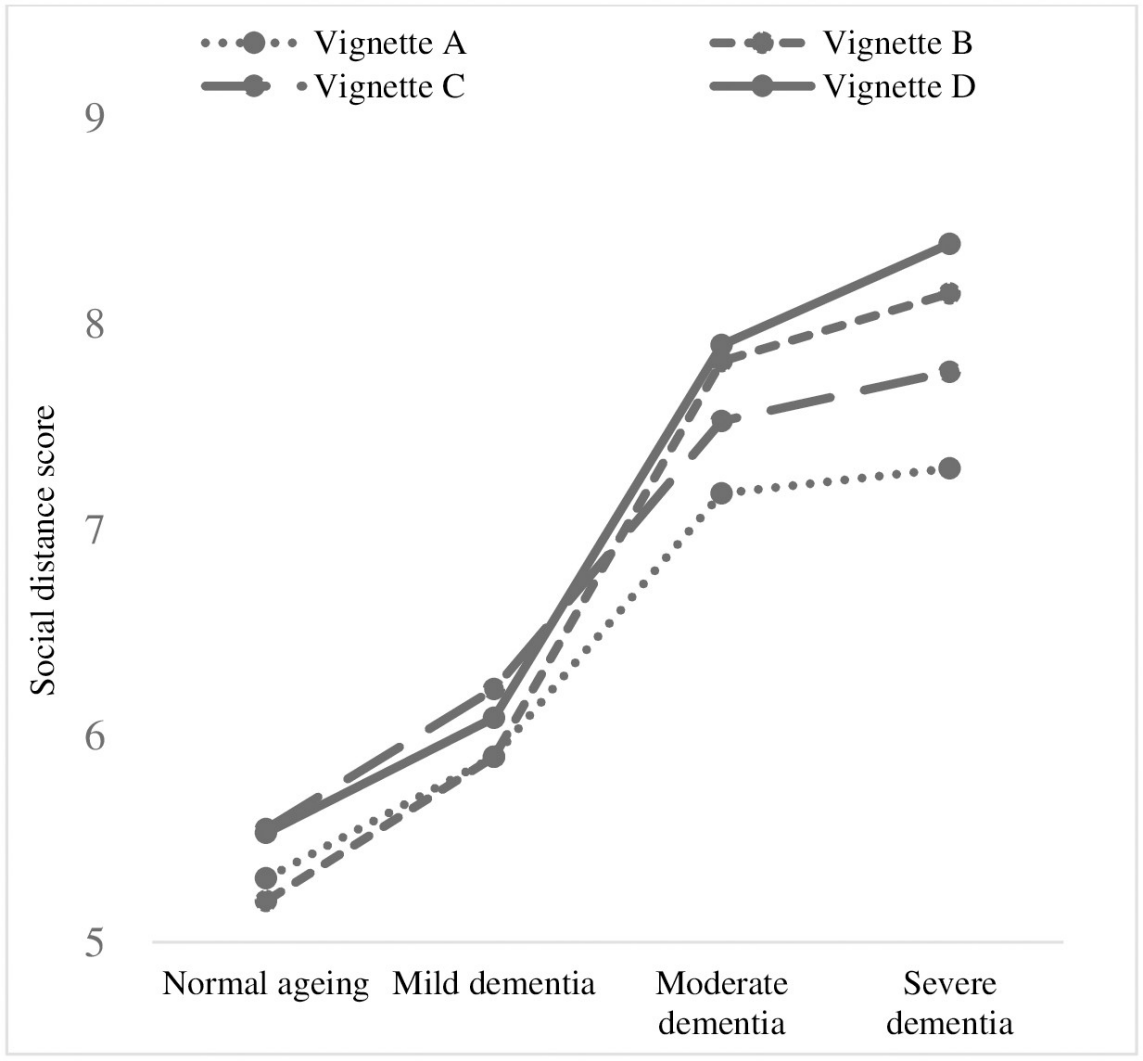
Vignette-specific changes in social distance by clinical stage of dementia. Social distance increases with the severity of dementia. At the moderate stage of dementia, irrespective of the living arrangement, social distance was larger for person living with dementia exhibiting behavioral and psychological symptoms of dementia. At the moderate and severe stage of dementia, the smallest social distance was observed for PLWD living with their family without BPSD, whereas the largest social distance was towards those living alone and exhibiting BPSD.

Fig 1 shows the vignette-specific changes in social distance by clinical stage of dementia. Social distance is greater in the more advanced stages of dementia. According to the results from Bonferroni multiple comparisons (Table 3), at the normal ageing and mild stages of dementia, social distance remained consistent with no significant differences observed across all vignettes.

**Table 3:** Results of Bonferroni multiple comparisons.

		Mean difference	95% CI		p value
**Moderate dementia**					
Vignette A	Vignette B	-0.64	-1.09	-0.18	0.002
	Vignette D	-0.72	-1.17	-0.26	< .001
**Severe dementia**					
Vignette A	Vignette B	-0.86	-1.32	-0.39	< .001
	Vignette C	-0.48	-0.94	-0.01	0.040
	Vignette D	-1.09	-1.56	-0.62	< .001
Vignette C	Vignette D	-0.62	-1.08	-0.15	0.003

At the moderate stage of dementia, social distance did not differ significantly between individuals exposed to Vignette A (living with her husband, without BPSD) and those exposed to Vignette C (living alone, without BPSD). Similarly, no significant difference in social distance was noted between those exposed to Vignette B (living with her husband, with BPSD) and those exposed to Vignette D (living alone, with BPSD). However, a notable finding was that individuals exposed to Vignette A displayed significantly higher social distance than those exposed to Vignette B (95% CI; -1.09, -0.18, p = 0.002) and Vignette D (95% CI: -1.17, -0.26. p<0.001). In summary, at the moderate stage of dementia, irrespective of the living arrangement, social distance was higher for PLWD exhibiting BPSD

At the severe stage of dementia, there was no significant difference in social distance between individuals exposed to Vignette B (living with her husband, with BPSD) and those exposed to Vignette C (living alone, without BPSD). However, individuals exposed to Vignette B (95% CI: -1.32, -0.39, p<0.001), Vignette C (95% CI: -0.94, -0.01, p = 0.004), and Vignette D (95% CI: -1.56, -0.62, p<0.001) displayed significantly higher social distance compared to those exposed to Vignette A (living with her husband, without BPSD). In addition, the social distance observed for individuals exposed to Vignette D (living alone, with BPSD) was significantly higher than for those exposed to Vignette C (living alone, without BPSD) (95% CI: -1.08, -0.15, p = 0.003). In summary, at the severe stage of dementia, the social distance towards PLWD living alone without BPSD was similar to those living with their husband and exhibiting BPSD. The lowest social distance was observed for PLWD living with their husband without BPSD, whereas the highest social distance was towards those living alone and exhibiting BPSD.

### Associated factors of preferences for social distance from PLWD in each vignette

To investigate the factors associated with social distance in each vignette, Vignettes A and D, which showed the largest variations in social distance, were examined. For each of these vignettes, the associated factors were explored by clinical stage of dementia. Multivariable linear regression analysis was performed separately for each vignette and by clinical stage of dementia with forced entry of the following factors: age, sex, years of education, marital status, living area, living arrangement, family income, social capital, perceived social support, subjective health, knowledge about dementia, social contact with PLWD, and cultural perspective measures (Table 4, placed at the end of the document).

**Table 4:** Factors associated with social distance in each vignette.

**Vignette A**															
	Mild dementia					Moderate dementia					Severe dementia				
	β	t	95%CI		p	β	t	95%CI		p	β	t	95%CI		p
Family income per year	-0.09	-2.11	-0.17	-0.01	0.035	-0.10	-2.10	-0.19	-0.01	0.036					
Social capital	-0.61	-5.93	-0.81	-0.41	<0.001	-0.66	-5.81	-0.88	-0.44	< .001	-0.63	-5.58	-0.85	-0.41	< .001
Perceived social support	-0.13	-2.79	-0.76	-0.13	0.005										
Knowledge about dementia	-0.14	-2.10	-0.28	-0.01	0.036										
Social contact with PLWD	-0.64	-2.69	-1.11	-0.17	0.007	-0.87	-3.30	-1.39	-0.35	0.001	-0.81	-3.10	-1.33	-0.30	0.002
**Vignette D**															
	Mild dementia					Moderate dementia					Severe dementia				
	β	t	95%CI		p	β	t	95%CI		p	β	t	95%CI		p
Sex	-0.61	-2.93	-1.03	-0.20	0.004										
Living area	0.61	3.00	0.21	1.02	0.003	0.47	1.98	0.00	0.94	0.048					
Social capital	-0.48	-5.34	-0.66	-0.30	<0.001	-0.36	-3.41	-0.57	-0.15	0.001	-0.37	-3.35	-0.58	-0.15	<0.001
Knowledge about dementia	-0.14	-2.23	-0.26	-0.02	0.026										
Social contact with PLWD	-0.60	-2.76	-1.03	-0.17	0.006	-1.10	-4.16	-1.55	-0.56	<0.001	-1.11	-4.21	-1.64	-0.60	<0.001
Self-confidence/ assertiveness						-0.20	-3.09	-0.33	-0.07	0.002	-0.18	-2.69	-0.32	-0.05	0.007
Social harmony/ adaption						0.10	2.34	0.02	0.19	0.020	0.13	2.75	0.04	0.22	0.006

Abbreviations: PLWD, people living with dementia

#### Vignette A

Possession of social capital (p<0.001) and having experience of social contact with PLWD (p = 0.001–0.007) were independently associated with lower social distance across all dementia stages. In the mild stage of dementia, high perceived social support (95% CI: -0.76, -0.13, p = 0.005) and having knowledge about dementia (95% CI: -0.28, -0.01, p = 0.036) were associated with lower social distance, but these associations were not observed in the moderate or severe stages. Having family income above average was associated with lower social distance in mild (95% CI: -0.17, -0.01, p = 0.035) and moderate dementia (95% CI: -0.19, -0.01, p = 0.036), but no association was found in the severe stage of dementia.

#### Vignette D

Possession of social capital (p≤0.001) and having experience of social contact with PLWD (p<0.001 to P = 0.006) were independently associated with lower social distance across all dementia stages.

In mild dementia, female sex (95% CI: -1.03, -0.20, p = 0.004) and having knowledge about dementia (95% CI: -0.26, -0.02, p = 0.026) were associated with lower social distance.

Living in a rural area was associated with lower social distance in mild (95% CI: 0.21, 1.02, p = 0.003) and moderate dementia (95% CI: 0.00, 0.94, p = 0.048), but not in severe dementia.

In moderate and severe dementia, traits related to individualism, such as high self-confidence/assertiveness, were associated with lower social distance (p = 0.002–0.007). Conversely, traits related to collectivism, such as social harmony/adaptation, were associated with higher social distance (p = 0.006–0.020).

#### Factors influencing social distance across dementia stages

- **Effect of Knowledge and Contact**
  Multivariable linear regression revealed that increased dementia-related knowledge was significantly associated with lower social distance toward PLWD, particularly in the mild stage, for both Vignette A (95% CI: -0.28, -0.01, p = 0.036) and Vignette D (95% CI: -0.26, -0.02, p = 0.026).
  Contact with PLWD was significantly associated with lower social distance at all dementia stages for both Vignette A (p = 0.001–0.007) and Vignette D (p < 0.001 to p = 0.006). These findings suggest that contact with PLWD is associated with lower social distance across all stages, while the influence of dementia-related knowledge is most significant in the mild stage.
- **Effect of Living Area**
  Multivariable linear regression revealed that living in a rural area was associated with lower social distance in Vignette D during mild (95% CI: 0.21, 1.02, p = 0.003) and moderate (95% CI: 0.00, 0.94, p = 0.048) stages of dementia, but this effect was not observed in the severe stage. Additionally, dementia-related knowledge levels did not differ significantly between urban and rural residents (95% CI: -0.32, 0.20, p = 0.63).
- **Effects of Cultural Perspectives**
  Traits related to individualism, such as self-confidence and assertiveness, were associated with lower social distance in Vignette D at the moderate (95% CI: -0.33, -0.07, p = 0.002) and severe (95% CI: -0.32, -0.05, p = 0.007) stages of dementia. In contrast, collectivistic traits related to social harmony and adaptation were associated with higher social distance in Vignette D at the moderate (95% CI: 0.02, 0.19, p = 0.020) and severe (95% CI: 0.04, 0.22, p = 0.006) stages of dementia.

## Discussion

### Social inclusion and exclusion in dementia care: Insights from Vignettes A and D

The results of the present study align with Werner’s previous study [28], showing that social distance increases with the severity of dementia. The association between BPSD and stigma also aligns with previous studies [44]. Additionally, some literature highlights the absence of family caregivers as a factor influencing stigma [45]. However, the present study introduces a new finding: the differences in social distance between Vignettes A and D highlight societal attitudes towards individuals with dementia.

Vignette A depicts a person living with her husband without BPSD, demonstrating the lowest social distance, which indicates higher social inclusion. In contrast, Vignette D shows a person living alone and exhibiting BPSD, associated with the highest social distance, indicating higher social exclusion. These findings reflect the broader cultural context in Japan, where there is a strong expectation for family members to assume caregiving responsibilities despite evolving social structures.

The Japanese law from the 8^th^ century mandated that older persons should be cared for by family members. If family members were unable to provide care, the community would take on this responsibility. From the 11^th^ century onward, the national welfare policy emphasized that caregiving for older persons should be a family or community affair, aligning with the Confucian’s principle of filial piety. This policy is reflected in the Law on Welfare for the Elderly enacted in 1963 [46]. The approach of relying on mutual support as an alternative to public support continued until 2000, when the Long-Term Care Insurance Law was implemented. However, the establishment of a new law does not immediately change people’s norm. In such a society, as Yang [45] noted, though stigma related to dementia can lower an individual’s status within both family and community, strong familial obligations often prevent outright rejection or abandonment. Consequently, PLWD who lack familial support face a higher risk of marginalization, and the presence of BPSD is often linked to rapid institutionalization [47], further marginalizing individuals from their communities.

Therefore, Vignette A represents the most socially inclusive scenario, whereas Vignette D represents the most marginalized. Participants’ responses reflect these dynamics, emphasizing the role of family presence and the impact of BPSD on social perceptions. These results align with the observed trend in Japan, where PLWD living alone and exhibiting BPSD are particularly vulnerable to marginalization or exclusion from the community [48] indicating that stigma may play a significant role in this phenomenon.

### Social inclusion and exclusion in dementia care: Insights from Vignettes B and C

Vignette A and Vignette B both depict a person living with her husband, with Vignette A showing a person without BPSD, while Vignette B shows a person exhibiting BPSD. In the moderate and severe stage of dementia, participants reported greater social distance toward the person in Vignette B compared to Vignette A. This suggests that BPSD is associated with greater social distance, regardless of living arrangements.

The label of BPSD may position PLWD as “less than whole” or “tainted”, embodying the very definition of stigma [49]. Such stigma is linked to negative outcomes, including reduced wellbeing, psychological distress, and a higher likelihood of community exclusion [44]. While managing BPSD is one approach, it is equally important to understand BPSD as an expression of loneliness [50]. Adopting this perspective may help reduce the stigma attached to BPSD, fostering more compassionate and inclusive attitudes toward PLWD.

In the severe stage of dementia, living alone appears to be a more salient factor for social distance than the presence of BPSD. This is reflected in Vignette C, where a person living alone without BPSD is associated with higher social distance than the person in Vignette A, who lives with her husband and does not exhibit BPSD. These findings suggest a pattern of heightened social distance toward PLWD who live alone compared to those living with others. Given that PLWD living alone are known to experience higher levels of loneliness than those living with others [51], the observed differences may reflect public stigma that contributes to social exclusion.

### Factors associated with social distance

Of the factors found to be independently associated with social distance toward PLWD in the present study, female sex [24], living in rural area [52, 53], high social capital [54], knowledge about dementia [24], experience of social contact with PLWD [24], and individualism [55] have also been identified as factors associated with social distance in previous studies. The study extends the understanding by differentiating these effects across dementia stages. Identifying stage-specific influences on social distance is a notable contribution that enhances targeted intervention strategies.

#### Effect of knowledge and contact

The findings confirm that education and social contact are essential for reducing stigma. While education is associated with lower stigma toward PLWD in general, it is insufficient on its own to address stigma in the moderate and severe stages (Table 4), highlighting the need for combined strategies. These findings align with existing literature emphasizing education and contact for stigma reduction [5, 56] but add nuance by addressing stage-specific effects.

However, evidence supporting education and contact as effective stigma-reduction methods remains limited [57, 58]. Moreover, close social proximity and shared experiences with PLWD may increase stigma, although this effect can be mitigated by increased knowledge [59]. To avoid unintended consequences, care must be taken to ensure that contact is guided by proper preparation, including interventions aimed to enhance access to and utilization of formal community care services [60]. Unstructured contact may inadvertently reinforce stigma [5].

#### Effect of living area

Previous studies suggest that stigma toward dementia is often stronger in rural communities [53, 61], though some have found no difference [54]. The present study reveals a more nuanced social dynamic depending on the stage of dementia. While rural communities may initially be inclusive, their inclusiveness has a threshold beyond which it tends to diminish. This dual nature of rural communities—both inclusive and exclusive—was also noted in our previous study on depopulated areas in Japan [62]. The findings highlight the stage-dependent nature of social distance in rural areas. While rural communities may be more inclusive toward individuals with mild dementia, including those with BPSD and no live-in family caregivers, this inclusiveness has a threshold. As dementia advances to the moderate and severe stages, rural and urban areas show no significant differences in social distance.

As Freeman [53] and Bacsu [63] pointed out, heightened stigma may result from a lack of dementia-related knowledge, which has historically been more pronounced in rural areas due to limited educational opportunities [64]. This study, however, found comparable knowledge levels among urban and rural residents, potentially mitigating the traditional stigma seen in rural areas.

#### Effects of cultural perspectives

This study explored the relationship between social distance and cultural perspectives, building on previous research [55] that examined the associations between stigma toward mental health conditions and individualism-collectivism. Previous findings indicate that collectivism is positively associated with stigma, while individualism is negatively associated with it.

The present study highlights the importance of cultural context in understanding social distance toward PLWD. Among individualistic traits, self-confidence and assertiveness are specifically associated with reduced social distance, while among collectivistic traits, social harmony and adaptation are associated with higher social distance. These findings suggest that fostering a balance between self-confidence and social flexibility may be key to effectively reducing stigma.

### Implication

Since the formulation of the “10-Year Plan for Understanding Dementia and Building Communities” in 2005, public awareness campaigns have primarily focused on educating people about dementia. The results of the present study suggest that such education has been effective in reducing stigma toward people with mild dementia. However, the findings also indicate that additional strategies are needed to reduce stigma toward individuals with moderate or advanced dementia, especially those living alone with BPSD with whom it may be challenging to interact with in daily life.

### Limitations of the study

A limitation of this study is the potential contamination of social distancing responses by social desirability bias [18]. Participants might underreport stigma to conform to socially desirable responses. Fiske [10] suggests that expressed attitudes may differ from actual behaviors. This study might have captured, in part, perceptions of the acceptability of expressing prejudice rather than stigma itself. Nonetheless, the experimental vignette method provided distinct social distance responses across different vignettes and dementia stages, indicating that social distance and its associated factors vary according to clinical stages, symptoms, and living arrangement.

### Future perspectives

Responses to individuals with dementia may vary across locations due to local social determinants of stigma. Given that this study was conducted in Japan, comparative studies in diverse cultural settings are necessary to generalize these findings and develop universal strategies for reducing stigma.

## Conclusions

The present study showed that social distance is greater toward PLWD who live alone and exhibit BPSD than toward those who live with family and/or do not show BPSD, indicating a higher risk of exclusion for the former. In addition, factors affecting social distance towards PLWD vary by the clinical stage of dementia. Increased knowledge about dementia reduces social distance toward PLWD, but this effect is mostly limited to the mild stage of dementia. In contrast, opportunities for social contact with individuals with dementia are crucial for reducing social distance at all stages. The findings suggest that, though education is associated with lower social distance toward PLWD in the mild stage, direct social contact is essential for achieving lower social distance toward PLWD in the moderate and severe stages.

## Supporting information

S1 Appendix (DOCX)
