# Supplementary material for: Effects of clinical stage, behavioral and psychological symptoms of dementia, and living arrangement on social distance towards people with dementia
Source: PLoS One. 2025 Jan 22;20(1):e0317911. doi: 10.1371/journal.pone.0317911 (PMC11753676; doi:10.1371/journal.pone.0317911)
Supplement: S1 Appendix — (DOCX) [file pone.0317911.s001.docx]

**S1** Vignettes

The vignettes were written in Japanese and included the following elements.

*Ms. A and Ms. B are 80-year-old women living with their husbands.*

*The following four scenarios illustrate how their situations evolve over time.*

Scenario A, B- 1

- Ms. A(B) sometimes goes shopping without her purse and goes back home to get it.
- The other day while cooking, she put too much spice in the pan and ruined her meal.
- Despite occasional forgetfulness, Ms. A(B) maintains a sense of independence, effortlessly dressing herself and attending to her personal needs.
- Her relationship with her husband remains strong, and she can talk about her mistakes freely to him.
- Ms. A interacts with her neighbors and participates with them in a gymnastics club for senior citizens.

Scenario A, B – 2

- Ms. A(B) was diagnosed with dementia, and she now sees her doctor regularly.
- Her husband sometimes assists with household tasks and her personal needs.
- Although her dressing, bathing, and toileting are still independent, her daily routines are now characterized by repetition and confusion.
- She cannot remember what happened a little while ago, and talks about the same things repeatedly.
- It takes her more time than before to prepare meals.
- She often buys items that are already in the refrigerator.
- Her husband accompanies her when she goes out because she has gotten lost in unfamiliar places.
- While she continues to interact with her neighbors, she is no longer a member of the gymnastics club.

Scenario A-3

- Ms. A often forgets what she was about to do. She unfolds the clothes she has already folded, then starts to fold them again.
- She can’t keep track of what she was doing. She forgets that she was cooking, and has burned food in the pan several times. Subsequently, she has stopped cooking.
- She often gets confused about which clothes to put on first.
- She needs assistance in her daily living.
- Although her husband is willing to help her, the inability to communicate with her leads to frustration and irritation for both of them.
- An incident of urinary incontinence prompted her to withdraw from social engagements.
- She now utilizes long-term care insurance services (home care services and senior day care center) several times a week.

Scenario A-4

- Ms. A spends much of her time in a daze, sometimes falling asleep even while eating.
- She speaks very little, and along with a decrease in her physical activities, there is a decrease in food intake and body weight.
- Urinary incontinence is a daily occurrence.
- She now requires assistance for her basic activities of daily living.
- She now utilizes long-term care insurance services (home care services and senior day care center) every day. However, she remains peaceful.
- Her husband has withdrawn from social contact due to the fatigue he suffers from caring for his wife.

Scenario B-3

- Ms. B often gets confused about which clothes to put on first.
- She needs assistance in her daily living.
- Although her husband is willing to help her, the inability to communicate with her leads to frustration and irritation, causing her to reject his assistance.
- She forgets that she was cooking, and has burned food in the pan several times. Subsequently, her husband persuaded her to stop cooking altogether.
- An incident of urinary incontinence prompted her to withdraw from social engagements.
- She now utilizes long-term care insurance services (home care services and senior day care center) several times a week.
- Several times a day, Ms. B becomes concerned about her money and begins to look around for her purse, accusing her husband of stealing from her. As this happens every day, her husband grows tired of being accused and confides in their neighbors.

Scenario B-4

- Ms. B often gets confused about which clothes to put on first.
- She needs assistance in her daily living.
- Although her husband is willing to help her, the inability to communicate with her leads to frustration and irritation, causing her to reject his assistance.
- Recently, the couple have quarreled because Ms. B insisted on cooking despite her husband’s instructions not to do so.
- There have been instances where Ms. B couldn’t find her way home after going out alone. One day, a neighbor found her wandering alone after dark. Her husband now feels compelled to accompany her at all times and is unable to rest, even for a moment.
- Her husband has decided to increase the utilization of long-term care services.

*Ms. C and Ms. D are 80 year-old women living alone. Their daughters reside an hour’s drive away. The following four scenarios illustrate how their situations evolve over time.*

Scenario C, D-1

- Ms. C(D) sometimes goes shopping without having her purse and goes back home to get it.
- The other day, while cooking she put too much spice in the pan and ruined her meal.
- Despite occasional forgetfulness, Ms. C(D) maintains a sense of independence, effortlessly dressing herself and attending to her personal needs.
- She interacts with her daughter several times a week.
- Ms. C(D) interacts with her neighbors and participates with them in a gymnastics club for senior citizens.

Scenario C, D – 2

- Ms. C(D) was diagnosed with dementia, and she now sees her doctor regularly.
- Her daughter comes to her home every weekend to assist with household tasks and her personal needs.
- Although her dressing, bathing, and toileting are still independent, her daily routines are now characterized by repetition and confusion.
- She cannot remember what happened a little while ago, and talks about the same things repeatedly.
- It takes her more time than before to prepare meals.
- She often buys items that are already in the refrigerator.
- She goes out with her daughter because she has gotten lost in unfamiliar places.
- While she continues to interact with her neighbors, she is no longer a member of the gymnastics club.

Scenario C-3

- Ms. C often forgets what she was about to do.
- She unfolds the clothes she has already folded, then starts to fold them again.
- She can’t keep track of what she was doing.
- She forgets that she was cooking, and has burned food in the pan several times. Her daughter subsequently advised her to stop cooking and has arranged a food delivery service for seniors.
- She often gets confused about which clothes to put on first.
- She needs assistance in her daily living, and utilizes long-term care insurance services (home care services and senior day care center) several times a week.
- Although her daughter is willing to help her, the inability to communicate with her leads to frustration and irritation for both of them.
- An incident of urinary incontinence prompted her to withdraw from social engagements.

Scenario C-4

- Ms. C spends much of her time in a daze, sometimes falling asleep even while eating.
- She speaks very little, and along with a decrease in her physical activities, there is a decrease in food intake and body weight.
- Urinary incontinence is a daily occurrence.
- She now requires assistance for her basic activities of daily living and utilizes long-term care insurance services (home care services and senior day care center) every day. However, she remains peaceful.
- It is difficult for her daughter to continue her job due to the fatigue she suffers from caring for her.

Scenario D-3

- Ms. D often gets confused about which clothes to put on first.
- She needs assistance in her daily living, and utilizes long-term care insurance services (home care services and senior day care center) several times a week.
- Although her daughter is willing to help her, the inability to communicate with her leads to frustration and irritation for both of them, causing her to reject her assistance.
- She forgets that she was cooking, and has burned food in the pan several times. Subsequently, her daughter persuaded her to stop cooking altogether.
- An incident of urinary incontinence prompted her to withdraw from social engagements.
- She now utilizes long-term care insurance services (home care services and senior day care center) several times a week.
- Several times a day, Ms. D becomes concerned about her money and begins to look around for her purse. She calls her daughter at work and accuses her of stealing from her. As this happens every day, her daughter grows tired of being accused by her mother and confides in her friends.

Scenario D-4

- Ms. D often gets confused about which clothes to put on first.
- She needs assistance in her daily living, and utilizes long-term care insurance services (home care services and senior day care center) every day.
- Although her daughter is willing to help her, the inability to communicate with her leads to frustration and irritation for both of them. causing her to reject her assistance.
- Recently, they have quarreled because Ms. D insisted on cooking despite her daughter’s instructions not to.
- There have been instances where Ms. D couldn’t find her way home after going out alone. One day, a neighbor found her wandering alone after dark. She was collected by the police and her daughter had to pick her up. Her daughter worries that this will happen again, which means she is unable to rest.
